# Broadband and high-efficiency polarization conversion with a nano-kirigami based metasurface

**DOI:** 10.1038/s41598-023-34590-1

**Published:** 2023-05-08

**Authors:** Xing Liu, Xiaochen Zhang, Weikang Dong, Qinghua Liang, Chang-Yin Ji, Jiafang Li

**Affiliations:** Key Lab of Advanced Optoelectronic Quantum Architecture and Measurement (Ministry of Education), Beijing Key Lab of Nanophotonics and Ultrafine Optoelectronic Systems, School of Physics, Beijing Institute of Technology, Beijing, 100081 China

**Keywords:** Nanoscale devices, Optoelectronic devices and components, Micro-optics

## Abstract

Nano-kirigami metasurfaces have attracted increasing attention due to their ease of three-dimension (3D) nanofabrication, versatile shape transformations, appealing manipulation capabilities and rich potential applications in nanophotonic devices. Through adding an out-of-plane degree of freedom to the double split-ring resonators (DSRR) by using nano-kirigami method, in this work we demonstrate the broadband and high-efficiency linear polarization conversion in the near-infrared wavelength band. Specifically, when the two-dimensional DSRR precursors are transformed into 3D counterparts, a polarization conversion ratio (PCR) of more than 90% is realized in wide spectral range from 1160 to 2030 nm. Furthermore, we demonstrate that the high-performance and broadband PCR can be readily tailored by deliberately deforming the vertical displacement or adjusting the structural parameters. Finally, as a proof-of-concept demonstration, the proposal is successfully verified by adopting the nano-kirigami fabrication method. The studied nano-kirigami based polymorphic DSRR mimic a sequence of discrete bulk optical components with multifunction, thereby eliminating the need for their mutual alignment and opening new possibilities.

## Introduction

Nano-kirigami metasurfaces^1^, which are composed of polymorphic subwavelength artificial nanostructures based on versatile shape transformation methods, not only bring new degree of freedom to the traditional three-dimensional (3D) nanomanufacturing, but also exhibit extraordinary potentials in the field of reconfigurable holograms^2,3^, fractal-dependent optical vortices^4^, Fano-resonant metamaterials^5–7^, reversal of circular dichroism^8,9^, and so on. Particularly, nano-kirigami^1,10,11^ could enable sophisticated shape changes from the two-dimensional (2D) precursors to 3D deformed nanostructures with vertical displacement, spatial bending, which provides a novel approach to manipulate the amplitude, phase, and polarization of electromagnetic waves in the microscale/nanoscale region. In this regard, the deformation of nano-kirigami structures have been induced by external stimuli such as pneumatic pressure^12^, mechanical compression^7,13^, electronic bias^14,15^, magnetic actuation^16^, and thermal expansion^17^. Among them, the global focused ion beam (FIB) irradiation induced tensile stress is approved as a facile strategy to induce permanent structural changes.

Meanwhile, due to the additional out-of-plane geometry, the versatile nano-kirigami geometries might enhance the performance of the metasurfaces. For example, polarization conversion exhibits splendid capacity of manipulating the polarization states in many scientific research systems. The major categories of conventional polarization converters include optical active crystals, liquid crystals, and the Faraday effect^18–25^. Nevertheless, these devices, suffer more or less from bulky volumes, low efficiency, narrow bandwidth, complex fabrication, etc. Metasurfaces provide an ideal platform to overcome these shortcomings. The polarization of light can be arbitrarily controlled by engineering subwavelength-thick meta-atoms of the metasurfaces. Taking advantage of the metasurfaces, broadband and high-efficiency polarization converter is promising to be realized with ultra-thin thickness.

Here, we report the generation of the broadband and high-efficiency polarization conversion in near-infrared wavelength by employing a nano-kirigami metasurface. By etching the local suspended nanofilms, metasurfaces with the polymorphic double split-ring resonators (DSRRs) and rich physical characteristics are realized. Both the simulations and experiments indicate that the 2D DSRR metasurfaces have two narrow bands of localized gap plasmons, which are merged to form a broadband and high-efficiency polarization conversion in the 3D upward deformed DSRR structures. The value of polarization conversion ratio (PCR) reaches more than 90% in the wavelength range from 1160 to 2030 nm. The demonstrated broadband and high-efficiency polarization conversion in such nano-kirigami based metasurfaces could be useful for the applications in ultracompact and integrated optoelectronic devices.

## Results and discussion

**Figure 1:**
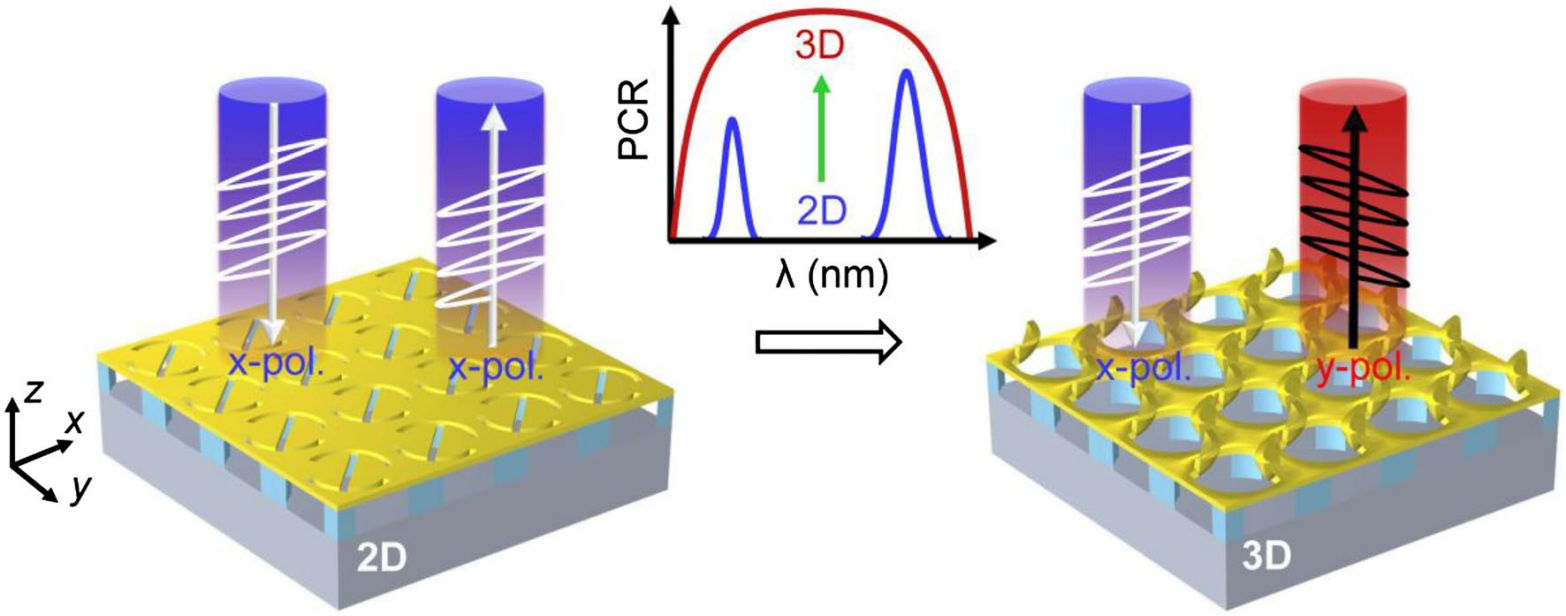
Schematic illustration of the broadband linear polarization conversion response of the nano-kirigami based DSRR metasurfaces. In the case of PCR spectra, the 2D structure shows two narrow bands while the 3D deformed structure possesses broadband and high-efficiency conversion.

Our proposed nano-kirigami is based on the classic DSRR metasurface, as illustrated in Fig. 1. The anisotropic DSRR structure is a typical open-loop structure that can be relative easily deformed compared with other close-loop geometries. It is composed of the 60-nm-thick top gold film, the intermediate SiO_2_ cuboid supporters with thickness of 300 nm and the bottom Si dielectric layer. Here, a thick enough silicon substrate can enhance the reflection at near-infrared wavelengths. The continuous curvature change of the circular shapes in the middle nano-slit can bring up stable structural deformation. Figure 1 schematically shows the operating principle in which the resonances of the two narrow bands from the 2D structure are merged to form a broadband polarization conversion in the case of 3D deformed structure.

**Figure 2:**
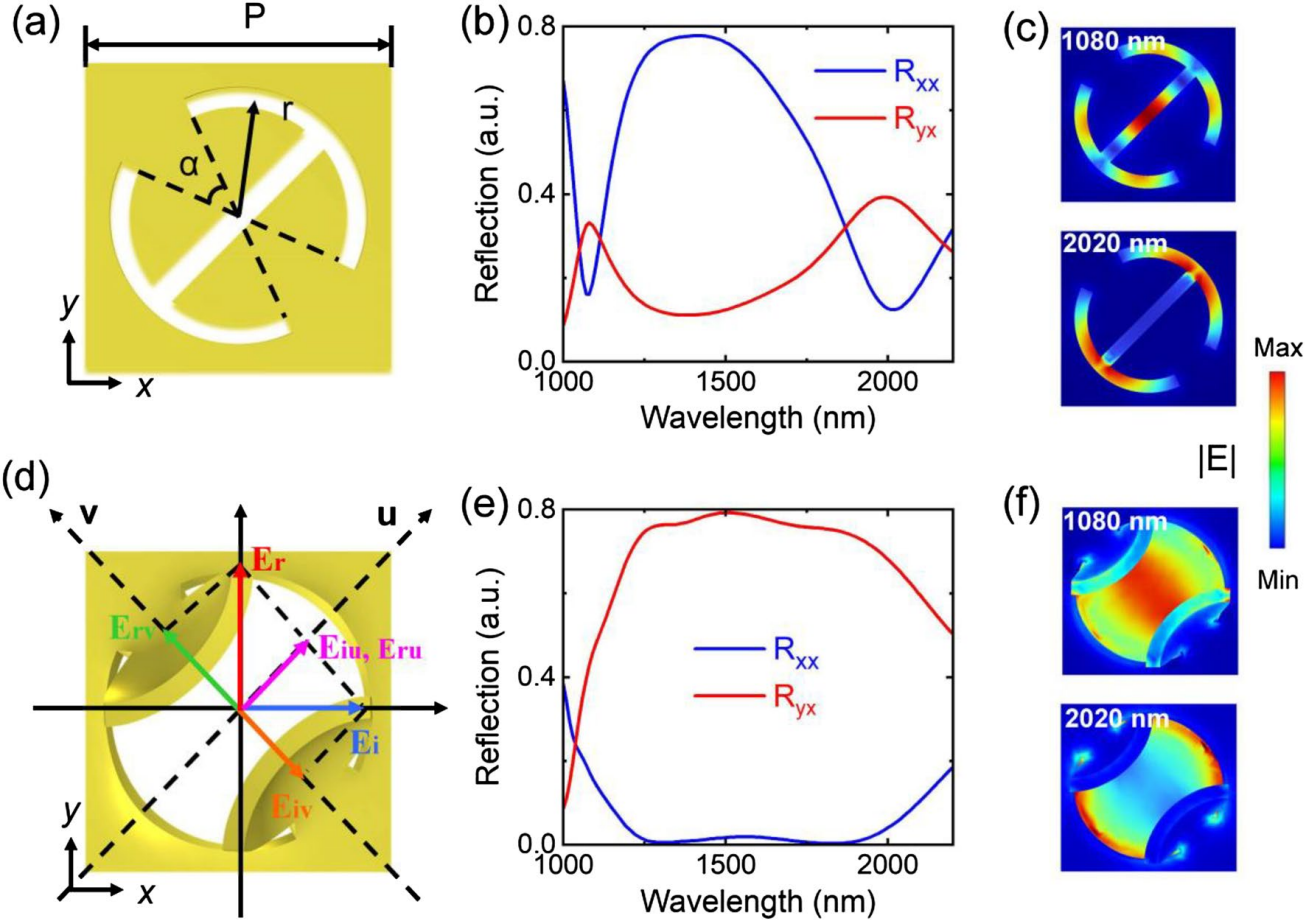
Schematic of unit cells of (**a**) the 2D structure and (**d**) the deformed 3D DSRR metasurfaces. (**b**) and (**e**) are the reflection spectra of the proposed 2D and 3D nanostructures under *x*-polarized normal incidence (cross-polarized reflection *R*_*yx*_, co-polarized reflection *R*_*xx*_). According to the resonance of the spectra, we plot the electric field distributions at the 1080, 2020 nm wavelength in figures (**c**) and (**f**).

As shown in Fig. 2a, the planar DSRR meta-atom is designed with 60 nm wide spiral air slit. The inner radius (*r*) of the spiral air slit is 0.31 μm. The angle (*α*) is 40° and the period (*p*) is 0.8 μm. Therein, the optical responses of the nanostructures are simulated by the finite element software COMSOL. The periodic boundary conditions are applied in the *xy* plane and the perfectly matched layer boundary condition is employed in the *z*-axis. Under *x* linearly polarized (LP) normal incidence, the planar DSRR exhibits two well-defined co-polarized and cross-polarized optical resonances in the near-infrared wavelength region, as shown in Fig. 2b. The co-polarization reflection* R*_*xx*_ is much larger than cross-polarized reflection* R*_*yx*_ in a bandwidth from 1200 to 2200 nm wavelengths, as shown in Fig. 2b. The two resonances in Fig. 2b are caused by the localized gap plasmons confined in the spiral air slit, as shown in the Fig. 2c.

By applying our previously developed bilayer stress distribution model^9^, the relationship between stress and height of the designed DSRR can be calculated and the desired nanostructures can be obtained. Under applying stress on the 2D DSRR nanostructure, the nanostructure opens the “door” along the *z* direction and causes the vertical deformation, as shown in Fig. 2d. The maximum height is 380 nm. In such a case, the out-of-plane degree of freedom of the structure is added and it can be used to tune the optical resonance of the structure. Obviously, under the deformed 3D state, the *R*_*xx*_ is much smaller than *R*_*yx*_ in a bandwidth from 1200 to 2200 nm, as shown in Fig. 2e. As a result, the *x*-polarized normal incident light is efficiently converted to its cross-polarized light after reflection. This is due to the fact that the mirror symmetry is broken in the deformed structures, which results in chiral optical responses and leads to the changes in the light-matter interaction. Meanwhile, the 3D nanostructures disrupt the original localized plasmon resonance modes, and the resonant wavelength of the two narrowband resonances in Fig. 2b are superimposed and thus broadened the spectral line. Figure 2f shows the electric field distributions of the stereo nanostructure, in which the localized surface plasmons modes are disappeared. Here, oblique light incident on the nanoslits will produce reflected light with different diffraction angles. For incident light with oblique incident angle of less than 10 degree, the PCR spectra of the structures change slightly, while too large incidence angle seriously affected the results (as shown in Supplementary Fig. S1).

In order to gain physical insight of the principle of polarization conversion, we decompose a *x-*LP normal incident light ***E***_*i*_ into two components of*** E***_*iu*_ and ***E***_*iv*_ in the *u*- and *v*- directions as sketched in Fig. 2d.

1 $${\varvec{E}}_{i} = ({\varvec{u}}E_{iu} + {\varvec{v}}E_{iv} )$$

and the electric component of the reflected wave is written as

2 $${\varvec{E}}_{{\varvec{r}}} = ({\varvec{u}}E_{ru} + {\varvec{v}}E_{rv} ) = ({\varvec{ur}}_{{\varvec{u}}} E_{iu} + {\varvec{vr}}_{{\varvec{v}}} E_{iv} ),$$

where ***r***_***u***_ = *r*_*u*_*e*^*iφu*^ and ***r***_***v***_ = *r*_*v*_*e*^*iφv*^ are the reflection coefficients in *u*- and *v*-directions respectively. Owing to the anisotropic characteristic of DSRR metasurfaces, there is a difference in the propagation phase of the components in the *u*- and *v*- direction of the polarized light, as Δ*φ* =|*φ*_*u*_ − *φ*_*v*_|. If the magnitudes and phase of the reflection coefficients satisfy the condition *r*_*u*_ = *r*_*v*_ and Δ*φ* = 180°, Eq. (2) becomes

3 $${\varvec{E}}_{{\varvec{r}}} = ({\varvec{y}}E_{r} e^{i\varphi u} ).$$

In our case, the conditions for obtaining efficient polarization conversion can be obtained by out-of-plane deformation of the structure.

The PCR is used to evaluate the performance of the proposed DSRR, which is defined as^26,27^:

4 $${\text{PCR}} = R_{yx} /(R_{xx} + R_{yx} )$$

**Figure 3:**
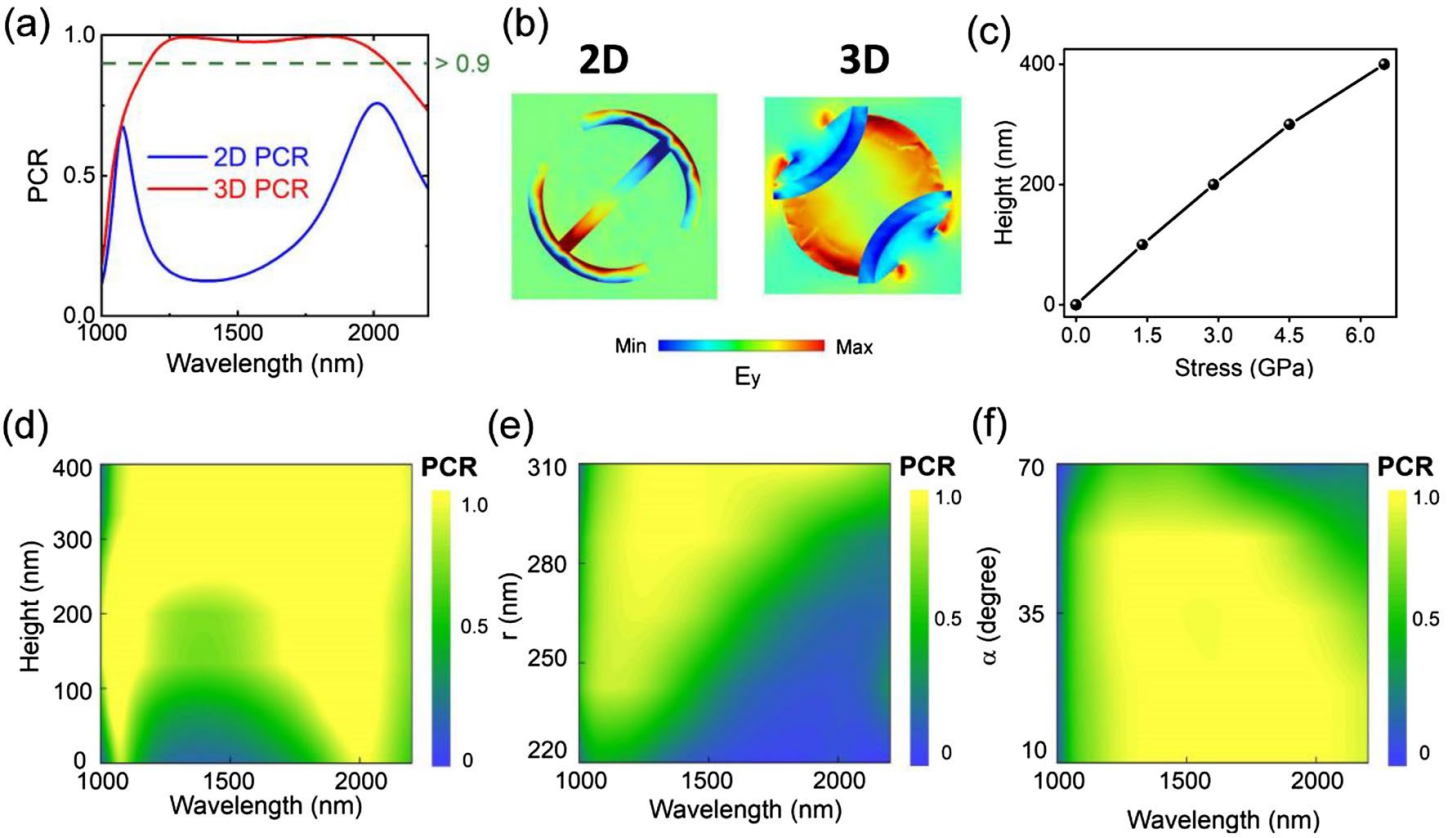
(**a**) The PCR spectra and (**b**) corresponding *E*_*y*_ distributions in *xy* plane at the 1500 nm of the proposed 2D and 3D metasurfaces. (**c**) Calculated relationship between pre-load stress and the vertical height of 3D DSRR nanostructures. In order to acquire the optimized performances, the PCR responses of the 3D nano-kirigami metasurface with respect to variation of (**d**) vertical height, (**e**) radii (*r*), (**f**) middle radian angle (*α*) are explored.

Figure 3a plots the PCR of the 2D DSRR nanostructure and 3D nanostructure. It is obvious in the 2D case that there are two narrow bands with PCR peak values of 0.67 and 0.75 at the resonance wavelengths of 1080 and 2020 nm, respectively. Meanwhile, the PCR of 2D state is very low from 1250 to 1750 nm wavelengths. Very interestingly, for the 3D deformed DSRR nanostructure, the PCR is larger than 0.9 in a broad wavelength range from 1200 to 2200 nm.

The PCR can also be understood from the distribution of *y*-component electric field *E*_*y*_ in the *xy* plane, as shown in Fig. 3b. It can be seen that the *E*_*y*_ presents odd and even symmetric about the diagonal *v*-axis and *u*-axis at 1500 nm wavelength for the 2D DSRR nanostructure, respectively. In such a case, the *E*_*y*_ in the far field is very small and thereby the PCR is very low. However, the *E*_*y*_ presents even symmetric about the coordinate origin of the *xy* plane at 1500 nm wavelength for 3D nanostructure. Thus, the *E*_*y*_ in the far field is very large and thereby the PCR is very high.

The performances of the 3D DSRR is also sensitive to the parameters and geometry shapes. Figure 3c shows the relationship between pre-load stress and the vertical height of 3D DSRR nanostructures. The results in Fig. 3d clearly show that the value and bandwidth of PCR increase with height. This directly highlights the importance of out-of-plane degrees of freedom in tuning the optical response. Based on sweeping different *r* and *α* parameters while keeping height fixed at 380 nm, simulation results of the corresponding reflected PCR amplitudes and bandwidth are plotted in Fig. 3e,f. For the 3D deformed DSRR, increasing the duty ratio of the nanostructure or reducing the middle radian angle *α* is the helpful way to improve PCR and expand bandwidth. For the 2D DSRR, the different structural parameters *r* and *α* shift the operating wavelength with a small bandwidth perturbation of the polarization conversion, as plotted in Fig. S2 of Supplementary Information. Based on these studies, we chose the structure parameters of *r* = 310 nm and *α* = 40° for experimental preparation.

**Figure 4:**
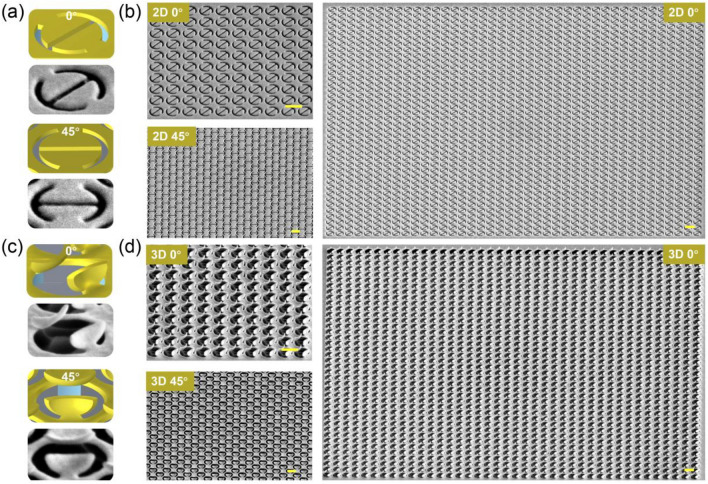
The experimental demonstration of the simulated DSRR nanostructure. (**a**) and (**c**) Schematic and SEM images of the unit cell of (**a**) 2D and (**c**) 3D DSRRs viewed at perspective angles of 0° and 45°, respectively. (**b**) and (**d**) SEM images of the corresponding 2D and 3D deformed nano-kirigami metasurfaces under different perspective view angles. Scale bars: 1 μm.

For experimental demonstrations, the individual DSRR meta-atom can be directly formed by using the high-dose focused ion beam (FIB) irradiation, as shown in Fig. 4a. After the wet etching process, the middle SiO_2_ layer is etched away to form the locally suspended 2D DSRR array. Figure 4b shows the SEM images of 2D DSRR array under the view angles of 0° and 45°. The overall size of the fabricated metasurface is 25 μm × 25 μm. Subsequently, the global low-dose FIB irradiation is employed to deform the 2D patterns into 3D metasurfaces by the nano-kirigami principle^1,9,10^. Figure 4c,d show the individual and metasurfaces of deformed DSRR in different situations. It can be seen that the experimentally fabricated nanostructures are in good agreement with the theoretically designed structures.

**Figure 5:**
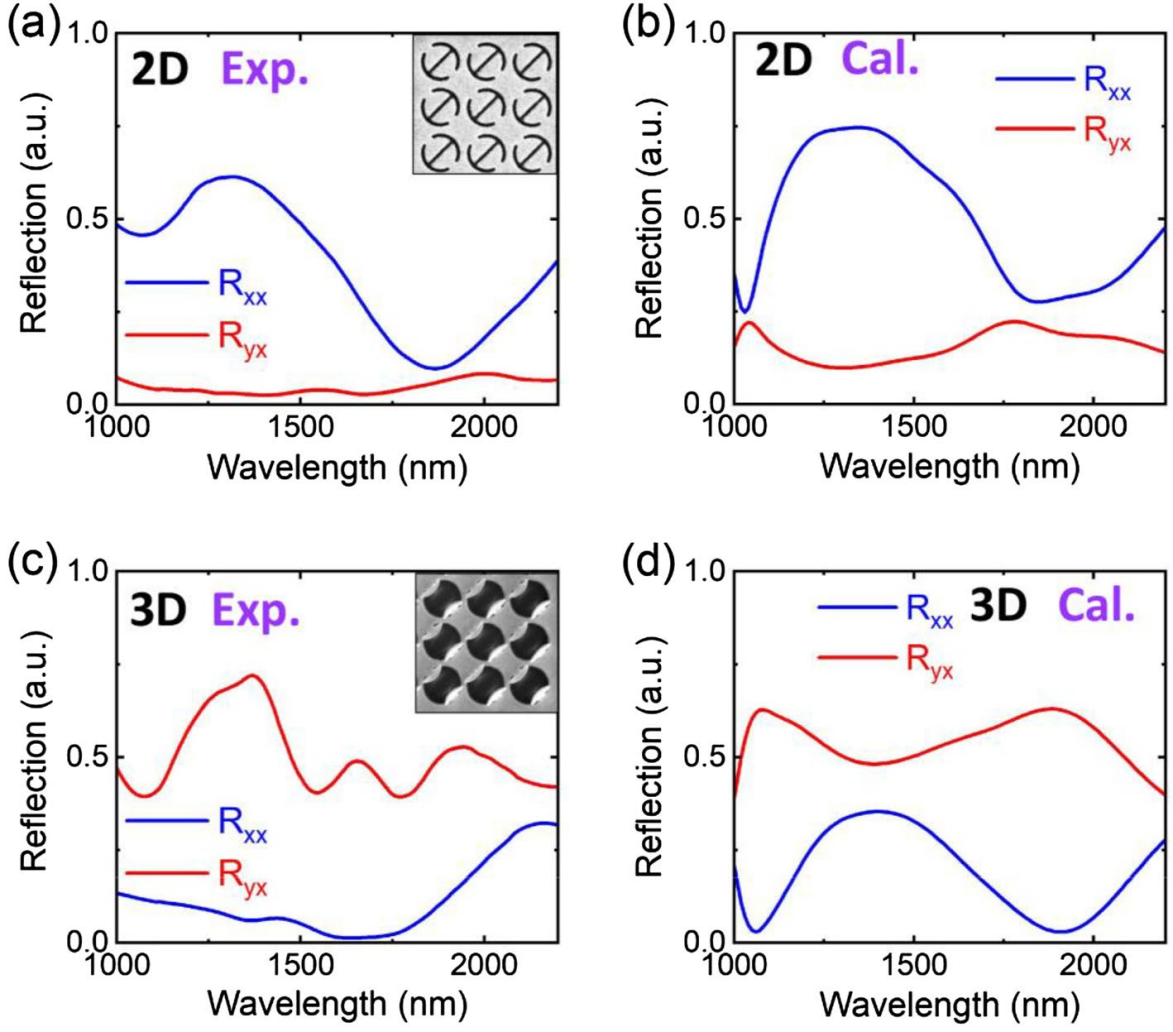
Comparison between experimental and theoretical results. (**a**), (**c**) Measured and (**b**), (**d**) simulated spectra of the 2D and 3D DSRR metasurfaces in the insets, respectively, under *x*-polarized light excitation.

The LP light for measurement can be obtained by adding a linear polarizer behind the light source and in front of the sample, as shown in Fig. S3 of Supplementary Information. The incident polarization of the light source is set along the *x* direction. Another linear polarizer is placed at the detection optical path along the *x* (*y*) direction to measure the co-polarized (cross-polarized) reflected signal *R*_*xx*_ (*R*_*yx*_). For the 2D metasurface, the results of experimental measurements and theoretical simulations are shown in Fig. 5a,b, respectively. The experimentally measured spectrum is basically consistent with the theoretical simulation. The Fig. 5a shows that experimentally measured *R*_*xx*_ is greater than *R*_*yx*_ over the entire spectral range. As shown in Fig. 5a, the *R*_*yx*_ is close to 0. Thus, it can be concluded that the PCR is very low for the 2D metasurfaces. The Fig. 5c,d are the results of the 3D DSRRs. It can be seen that the experimentally measured *R*_*yx*_ is greater than *R*_*xx*_ over the entire spectral range. The broadband cross-polarization reflection occurs in wavelengths from 1000 to 2200 nm. In such a case, the broadband and high-efficiency polarization conversion is achieved in the near-infrared wavelength region with the help of the out-of-plane deformation of the DSRR. The slight difference between the measurement (Fig. 5c) and simulation (Fig. 5d) is mainly caused by the material loss possibly induced under the implantation of Ga^+^ ions. After FIB irradiation, the irradiated area is slightly modified regarding surface roughness and refractive index, which affect the optical properties compared to the ideal material parameters in simulations^28^. Such metasurfaces with broadband polarization conversion avoid the complicated fabrication process of multilayered nanofabrication and offer great values in the polarization controlling devices^29–35^.

## Conclusion

In this work, we have utilized DSRR meta-atoms to realize high-efficiency and broadband linear polarization conversion in the near-infrared wavelength band based on the nano-kirigami principle. The fabricated 2D metasurface exhibits two resonance bands with reflection polarization orthogonal to the incident polarization direction, which are caused by localized surface plasmon in gaps. The out-of-plane deformation of the 3D DSRRs after nano-kirigami tune the responses of the localized surface plasmons. As a result, the operation bands are converted into broadband for the cross-polarized reflection, with high efficiency in the near-infrared wavelength region under LP incidence. The PCR is more than 90% in wavelength range from 1160 to 2030 nm (147–256 THz) in the case of the 3D structure, which is advantageous compared with other wideband polarization converters reported in^36–40^ (see Table 1 of Supplementary Information). Obviously, the proof-of-concept demonstration is successfully verified by adopting the nano-kirigami fabrication method. The polymorphic DSRR nanostructures derive a novel metasurface that is possible to replace bulk optical components with ultracompact and integrated optoelectronic configurations. For example, reconfigurable metasurfaces could be readily realized by introducing a bias voltage between the top metal and bottom conductive substrate, which can generate electrostatic force to dynamically tune the height of downward deformation (see Figure S4 of Supplementary Information). With the increase of the external bias voltages, the wavelength of the resonance modes can be tuned and in such a case, the operation wavelength and PCR of the polarization conversion can be dynamically engineered.

## Supplementary Information

Supplementary Information.

## Data Availability

All the data supporting the findings of this study are available within the article or from the corresponding authors upon reasonable request.
