# Supplementary material for: Broadband and high-efficiency polarization conversion with a nano-kirigami based metasurface
Source: Sci Rep. 2023 May 8;13:7454. doi: 10.1038/s41598-023-34590-1 (PMC10167358; doi:10.1038/s41598-023-34590-1)
Supplement: Supplementary file 1 — Supplementary Information. [file 41598_2023_34590_MOESM1_ESM.pdf]

Broadband and high-efficiency polarization conversion with a nano-kirigami based metasurface

Xing Liu, Xiaochen Zhang, Weikang Dong, Qinghua Liang, Chang-Yin Ji*, Jiafang Li*

Key Lab of Advanced Optoelectronic Quantum Architecture and Measurement (Ministry of Education), Beijing Key Lab of Nanophotonics & Ultrafine Optoelectronic Systems, School of Physics, Beijing Institute of Technology, Beijing 100081, China

** Corresponding authors: jcyinbit@bit.edu.cn; jiafangli@bit.edu.cn*

Supplementary Note 1:

Sample Fabrications: The samples are prepared by the focused ion beam lithography (a FIB/EBL dual-beam system, Helios G4 UC) and the hydrofluoric acid (HF) wet-etching. Using the focused ion beam under high dosage of >600 pC/μm^2 focused ion beam, the designed 2D patterns are milled on the gold nano-film. The free-standing gold nano-film supported by the SiO_2 pillars can be formed by precisely controlling the etching time of the sample immersed in the 40% HF solution for 3 minutes. Subsequently, the dose of FIB is adjusted to a relatively low value of 10-40 pC/μm^2, and the suspended 2D nano-structures are deformed into the 3D geometries by globally irradiating the entire metasurface. The relationship between structural deformation and the exposure dosage can be found in Ref. [9]

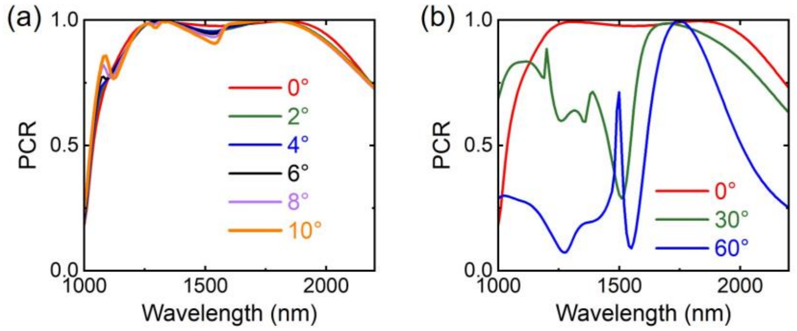

Fig. S1. The PCR spectra of the proposed 3D nano-structures under different incident angle.

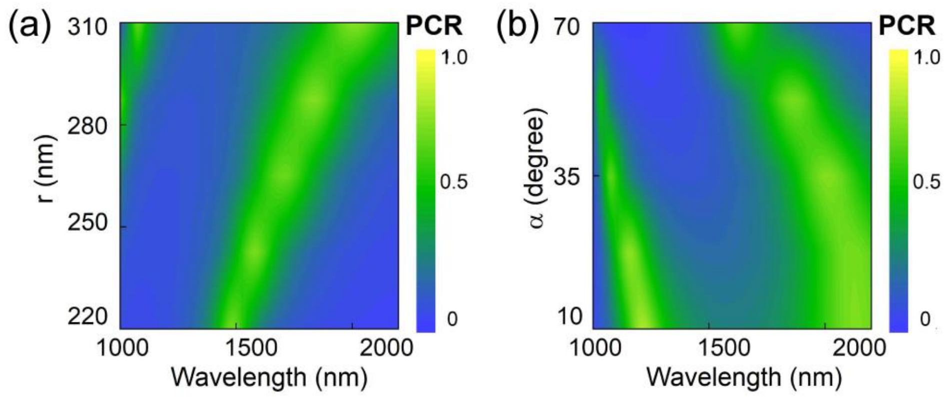

Fig. S2. The PCR responses of the 2D DSRR metasurfaces with respect to variation of (a) radii (r), (b) middle radian angle (α) are explored.

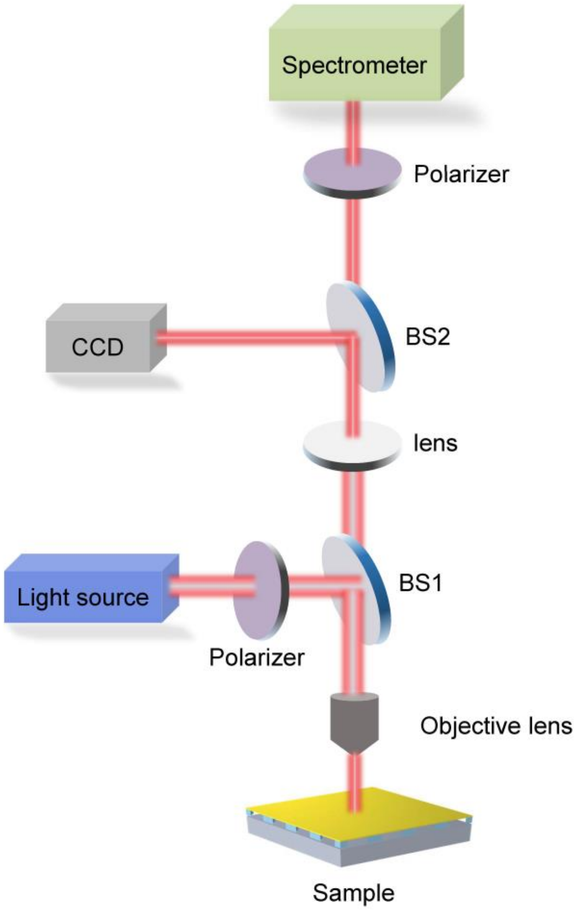

Fig. S3. Schematic illustration of the optical measurements setup.

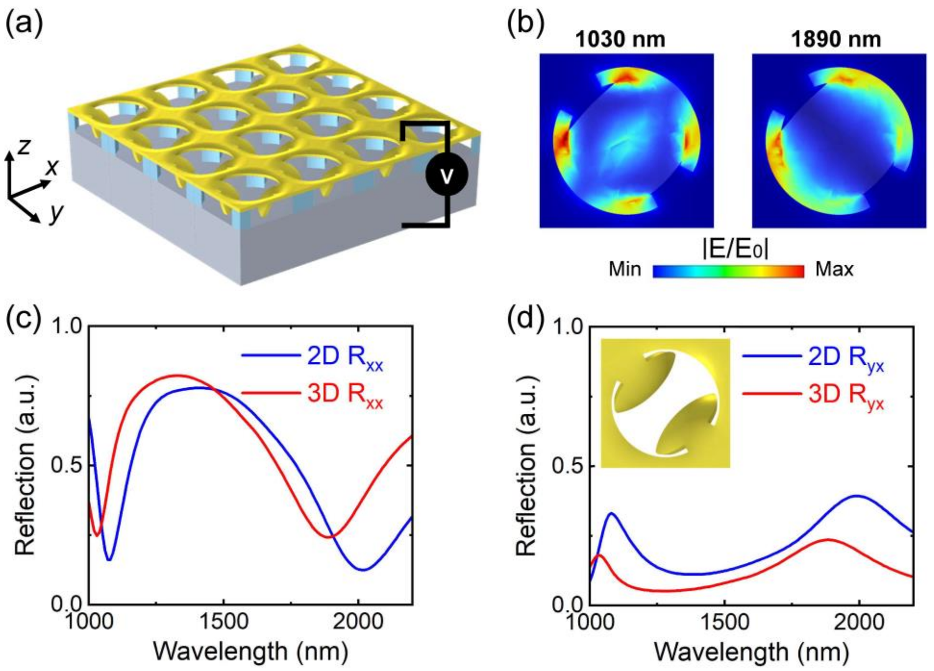

Fig. S4. Optical response of the exotic electromechanical nano-kirigami in calculations. (a) Schematic of the deformed 3D electromechanical nano-kirigami metasurface. For the 3D structure, the downward deformation height is -160 nm along the *z*-axis under the applied voltage of 280 V. (b) The electric field distributions of the 3D DSRR nanostructure at the two resonant wavelengths. Comparison between (c) 2D and (d) 3D deformed nano-kirigami metasurfaces in the case of the co-polarized and cross-polarized reflection, respectively.

Ref	Configurations	Frequency range (THz)	Highest efficiency	Published year
Jiang et al.	Au-dielectric-Au	>PCR 90%, 0.57 – 1.12	100%	2020
Qi et al.	Cu-dielectric-Cu	>PCR 90%, 2.04 – 5.33	100%	2020
Jiang et al.	Al-polyimide-Al-polyimide-Al	>PCR 90%, 0.73 – 2.24	99.5%	2021
Wei et al.	Cu-dielectric-Cu	>PCR 89%, 2.28 – 6.75	99%	2022
Tamim et al.	Al-GaAs-Al	>PCR 90%, 0.987 – 1.062, 0.442 – 0.537	100%	2022
This work	Au-SiO_2-dielectric	>PCR 90%, 147– 256,	100%	

Table 1. Comparison of proposed 3D DSRR nano-kirigami-based metasurface with recently reported[36-40] broadband polarization converters.
